# Hypocalcemia with Immune Checkpoint Inhibitors: The Disparity among Various Reports

**DOI:** 10.1155/2020/7459268

**Published:** 2020-06-06

**Authors:** Swarna Sri Nalluru, Paramarajan Piranavan, Ying Ning, Haritha Ackula, Ahmad D. Siddiqui, Nitin Trivedi

**Affiliations:** ^1^Department of Medicine, Saint Vincent Hospital, Worcester, Massachusetts 01608, USA; ^2^Department of Rheumatology, State University of New York, Syracuse, New York 13210, USA; ^3^Department of Hematology-Oncology, Saint Vincent Hospital, Worcester, Massachusetts 01608, USA; ^4^Department of Endocrinology, Saint Vincent Hospital, Worcester, Massachusetts 01608, USA

## Abstract

Immune-related adverse events affecting parathyroid function are rarely reported with immune checkpoint inhibitors (ICPIs). Activating calcium-sensing receptor antibodies causing autoimmune hypoparathyroidism with nivolumab was recently reported. KEYNOTE-189 and CHECKMATE-067 trials reported a 21–29% hypocalcemia event rate, but the etiology of hypocalcemia was not reported. A chart review was performed to study patients receiving ICPI from 2015 to 2018 at multiple sites affiliated with Saint Vincent Hospital. The study population was divided into two groups based on the presence or absence of calcium altering conditions or medications. True hypocalcemia incidence was calculated after correcting calcium for albumin from the initiation of ICPI to their last follow-up. Group 1 (*n* = 83) includes patients with no calcium altering conditions or medications. Group 2 (*n* = 98) includes patients on calcium supplements (*n* = 17), vitamin D (*n* = 44), bisphosphonates (*n* = 24), >stage IIIB chronic kidney disease (CKD) (*n* = 5), and bone metastasis (*n* = 38). Hypocalcemia events in Group 1 vs. Group 2 were 8.4% and 19.3%, respectively. Our entire study demonstrated 26.8% vs. 1.1% of Grade I vs. II hypocalcemia events. However, after correcting the calcium for albumin, hypocalcemia incidence was 0.56% (*n* = 1). No further workup was done to investigate the etiology as that patient passed away. Our data suggest that the true hypocalcemia incidence after using albumin-corrected calcium values is very low in patients receiving IPCI, even in the presence of calcium altering factors. The percentage of patients with hypocalcemia is much higher and similar to the KEYNOTE-189 and CHECKMATE-067 trials when serum calcium values without albumin correction are used. Thus, the higher reported incidence of hypocalcemia in these trials is likely due to the reporting of serum calcium without albumin correction.

## 1. Introduction

ICPIs are rapidly evolving targeted therapies (monoclonal antibodies) against immune checkpoint molecules, i.e., PD-1, PDL-1, and CTLA-4. While CTLA-4 regulates early T-cell proliferation mainly in lymph nodes, PD-1 primarily suppresses T-cell activation in an immune response in peripheral tissues. ICPIs block these specific molecules and regulate the T-cell-mediated antitumor activity. As the PD-1 pathway blockade results in activation of quiescent antitumor T cells and decreased self-tolerance, CTLA-4 pathway blockade results in reduced T regulatory cell-mediated immunosuppression and hyperproliferation. A dual pathway blockade causes enhanced antitumor immune response due to synergism [1]. Loss of self-tolerance and decreased T-cell regulatory response may produce several immune-related adverse events (IRAEs) through autoantibodies, autoreactive T cells, and enhanced cytokine expression, although the exact pathophysiology is not yet known [2]. IRAEs are organ-specific toxicities and vary with ICPI therapy type. They are most commonly noted in the skin followed by the gastrointestinal tract, liver, endocrine system, etc. In the endocrine system, thyroid, pituitary, pancreas, and adrenal glands are commonly affected, but the involvement of the parathyroid gland is rarely reported [2]. Our review of the literature showed a few published cases of hypocalcemia secondary to ICPI suggesting parathyroid involvement [3–6]. Recent reports demonstrated that activating calcium-sensing receptor antibodies are associated with the development of autoimmune hypoparathyroidism in a patient receiving nivolumab [3]. Several trials like KEYNOTE-189 and CHECKMATE-067 trials reported a 21–29% hypocalcemia event rate. Our study aimed at identifying the hypocalcemia incidence and severity in patients receiving ICPI at a single institution. We also intended to investigate hypoparathyroidism as the etiology in these patients, if hypocalcemia was detected.

## 2. Methods

A retrospective review of various types of cancer patients receiving PD-1 (pembrolizumab), PDL-1 (nivolumab), and CTLA-4 (ipilimumab) inhibitors between 2014 and 2018 was carried out at two sites (Reliant Medical Group Hematology/Oncology and the Saint Vincent Hospital Cancer Center) affiliated with the Saint Vincent Hospital in Worcester, Massachusetts. All of these patients received immunotherapy as an initial agent or transitioned later due to initial treatment failure or intolerance. Based on the standard treatment protocols for specific cancer, they were either on single or double immunotherapy agents. As we aimed at identifying the incidence of parathyroid-related hypocalcemia in cancer patients receiving ICPI, we extracted relevant data from this set of population.

According to our laboratory (LabCorp) parameters, true hypocalcemia is defined as the calcium levels less than 8.6 mg/dl after correcting for an abnormal serum albumin, and hypoalbuminemia is defined as albumin levels less than 3.5 mg/dl. Corrected calcium was estimated using Parent et al.'s formula, i.e., corrected calcium = (0.8 × (normal albumin − patient's albumin)) + serum calcium [7]. The incidence of hypocalcemia events was calculated from the initiation of immune checkpoint inhibitors until their last follow-up. Hypocalcemia was graded based on the CTCAE v 5.0 (common terminology criteria for adverse events) published by the National Cancer Institute. To identify the incidence of hypocalcemia attributed to hypoparathyroidism and minimize the bias, we identified the factors that could potentially affect the serum calcium levels. The factors that were recognized are medications (calcium supplements, vitamin D, and bisphosphonates) or medical conditions (preexisting diagnosis of hyperparathyroidism/hypoparathyroidism, vitamin D deficiency, coexisting hypomagnesemia, hyperphosphatemia, >stage III B CKD, parathyroidectomy, neck surgery/radiation therapy resulting in parathyroid abnormalities, paraneoplastic syndromes resulting in abnormal parathyroid hormone-related peptide (PTHrP) production, and cancerous lesions in bone) [8]. We subdivided our patient population into two groups based on the presence or absence of the abovementioned risk factors altering the serum calcium levels. Data extracted included age, gender, type of cancer, type and dosing of ICPI, factors affecting calcium levels, and relevant laboratory data (calcium, albumin, magnesium, phosphate, and creatinine clearance). Statistical analysis was done using SPSS software v. 21.0 (SPSS Inc., Armonk, NY, USA) to calculate the median age and IQR. The study protocol was approved by the local institutional review board.

## 3. Results

A total of 178 patients were included in our single-institution multicenter retrospective study. The baseline characteristics of our study population are shown in Table 1. The median age of our study population was 70 years (IQR = 78 − 62.5).

Group 1 (*n* = 83) was considered low-risk group for abnormal calcium events as it included patients with no serum calcium altering conditions or medications.

Group 2 (*n* = 98) was considered high-risk group for abnormal calcium events as it included patients with the presence of any of the abovementioned risk factors, i.e., calcium supplements (*n* = 17), vitamin D use (*n* = 44), bisphosphonates (*n* = 24), >stage IIIB CKD (*n* = 5), and metastatic lesions in bone (*n* = 38).

In Group 1 (*n* = 83), patients were on single agent (pembrolizumab (*n* = 25), nivolumab (*n* = 56), and ipilimumab (*n* = 2)) and combination (nivolumab-ipilimumab) immunotherapies (*n* = 3). Grade I hypocalcemia incidence was 8.4% (*n* = 7). After correcting the calcium to albumin, zero incidence of true hypocalcemia was reported.

In Group 2 (*n* = 98), 81.6% of patients were on single agent (pembrolizumab (*n* = 35), nivolumab (*n* = 44), and ipilimumab (*n* = 1)) and 4.08% on combination (nivolumab-ipilimumab) immunotherapies (*n* = 4). 18.4% of Grade I hypocalcemia (*n* = 18) and 1.02% of Grade II hypocalcemia (*n* = 1) incidence were reported. No Grade III or IV events were noted. The total incidence of hypocalcemia events noted in this group was 19.3%. True hypocalcemia incidence was only 1.02% (*n* = 1) as most of the hypocalcemia events normalized during the next follow-up or after correcting to albumin. Further workup to identify the etiology was not initiated as this one patient passed away.

Our entire study demonstrated 26.8% vs. 1.1% of Grade I vs. II hypocalcemia events. However, after correcting the calcium for albumin, only one patient, i.e., 0.56% of the entire study demonstrated true hypocalcemia.

Group 1 showed a total of 36.1% (*n* = 30) patients that were lost to follow-up (19 died, 6 treatment intolerant, 5 entered hospice, and 2 went to another hospital).

Group 2 showed a total of 26.5% (*n* = 26) patients that were lost to follow-up (14 died, 2 treatment intolerant, 6 entered hospice, and 4 went to another hospital).

**Figure 1:**
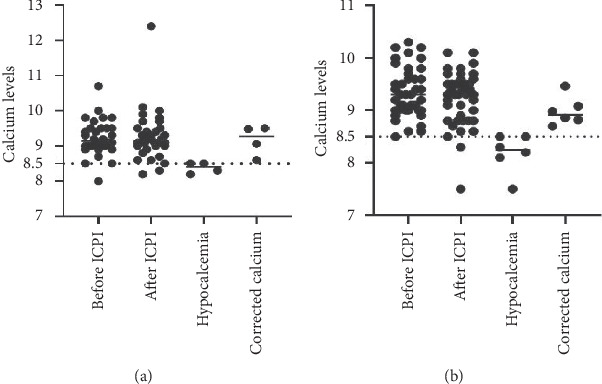
Hypocalcemia events in patients receiving pembrolizumab with (a) and without risk factors (b). *Y*-axis depicts the range of calcium levels. *X*-axis depicts several values. Before ICPI indicates the range of calcium levels before initiation of ICPI treatment. After ICPI indicates the range of calcium levels after ICPI treatment. Hypocalcemia indicates the low calcium levels noted during and after the treatment. Corrected calcium indicates the calcium levels after correcting the low calcium levels with albumin.

**Figure 2:**
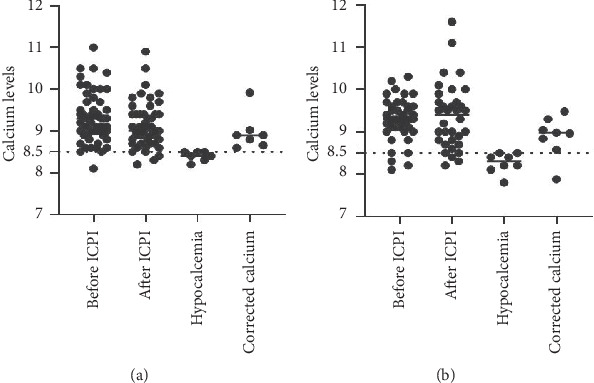
Hypocalcemia events in patients receiving nivolumab with (a) and without risk factors (b). *Y*-axis depicts the range of calcium levels. *X*-axis depicts several values. Before ICPI indicates the range of calcium levels before initiation of ICPI treatment. After ICPI indicates the range of calcium levels after ICPI treatment. Hypocalcemia indicates the low calcium levels noted during and after the treatment. Corrected calcium indicates the calcium levels after correcting the low calcium levels with albumin.

**Figure 3:**
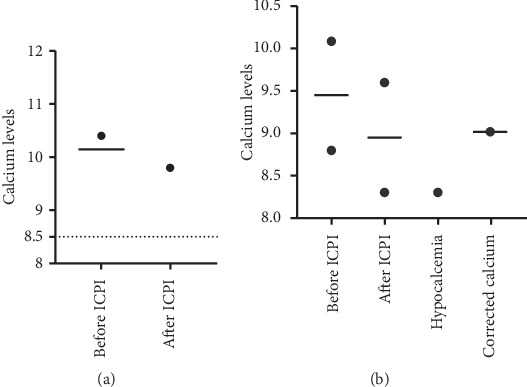
Hypocalcemia events in patients receiving ipilimumab with (a) and without risk factors (b). *Y*-axis depicts the range of calcium levels. *X*-axis depicts several values. Before ICPI indicates the range of calcium levels before initiation of ICPI treatment. After ICPI indicates the range of calcium levels after ICPI treatment. Hypocalcemia indicates the low calcium levels noted during and after the treatment. Corrected calcium indicates the calcium levels after correcting the low calcium levels with albumin.

**Figure 4:**
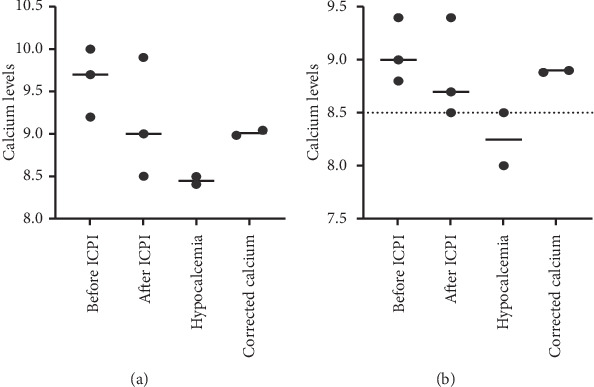
Hypocalcemia events in patients receiving nivolumab-ipilimumab with (a) and without risk factors (b). *Y*-axis depicts the range of calcium levels. *X*-axis depicts several values. Before ICPI indicates the range of calcium levels before initiation of ICPI treatment. After ICPI indicates the range of calcium levels after ICPI treatment. Hypocalcemia indicates the low calcium levels noted during and after the treatment. Corrected calcium indicates the calcium levels after correcting the low calcium levels with albumin.

Figures 1–4 demonstrate calcium levels before and after ICPI therapy in patients receiving various ICPI drug types.

## 4. Discussion

Calcium homeostasis is a tightly regulated process by the intestinal tract, kidneys, and bone and is mediated by several hormones like parathyroid hormone, calcitonin, and 1,25-dihydroxyvitamin D3. While majority (∼98%) of the total body calcium is present in the bone, only 1% is freely exchangeable with extracellular fluid (ECF). This circulating calcium in ECF exists as free ionized calcium (∼50%), protein-bound calcium—mainly albumin and to a smaller extent to globulins (40%), and complexed to anions such as bicarbonate, citrate, sulphate, phosphate, and lactate (10%) [9]. Ionized calcium accounts for the biologically active form of serum calcium and plays a crucial role in the calcium homeostasis [10–12]. Due to the limitations in direct measurement of ionized calcium, including lack of standardization, sample handling difficulties, and cost, total serum calcium measurement continues to be routinely employed [13]. However, the levels of total serum calcium fluctuate with serum protein levels as significant proportion of calcium binds to proteins, particularly albumin. To mitigate the errors from dependent factors like albumin on total calcium measurement, adjusted calcium levels are calculated using Parent et al.'s formula, especially in cases of hypoalbuminemia [7].

Analysis of our study population showed a significant number of patients with serum calcium altering factors such as bone metastases and >stage III B CKD and medications such as calcium supplements, vitamin D, and/or bisphosphonates, as mentioned in Table 1. To minimize the confounding effect from these variables, we categorized our patient population into two groups based on the presence or absence of factors mentioned above. Our study showed an incidence of 8.4% and 19.3% Grade I-II hypocalcemia events in Group 1 vs. Group 2, respectively. No Grade III-IV events were noted. Although we used CTCAE v5 criteria to prevent the disparity in grading hypocalcemia, it must also be noted that the lower normal of calcium levels vary with the laboratory parameters. Several clinical trials noted an incidence of all-grade hypocalcemia events with slight variation depending on the drug and its dosage. The package inserts of Keytruda and Opdivo report 27%, 13%, 21%, and 29% of all-grade hypocalcemia events with pembrolizumab, nivolumab, ipilimumab, and nivolumab-ipilimumab, respectively, with the data collected from KEYNOTE-158 and CHECKMATE-067. KEYNOTE-012 reported a maximum of 6.67% of not serious hypocalcemia events along with hypoalbuminemia of 6.67%. Although all-grade hypocalcemia events were noted with these drugs, no further details like persisting or transient hypocalcemia or corrected calcium levels for albumin were not provided.

To identify the exact incidence of low calcium levels in our study, we corrected the noted cases of hypocalcemia with their corresponding serum albumin levels due to the lack of data on ionized calcium levels on retrospective review and found that the incidence of true hypocalcemia was only 0.56% (*n* = 1). As the patient had passed away, no further workup was possible to identify the association of hypocalcemia with parathyroid abnormalities. The low incidence of hypocalcemia, irrespective of its etiology, implies a rare involvement of the parathyroid gland with ICPI in cancer patients, consistent with the rare incidence of autoimmune hypoparathyroidism in general population [14].

In contrary to Piranavan's reports of activated CaSR antibodies as a probable causation for ICPI-related autoimmune hypoparathyroidism, Trinh et al. suggested inflammation-mediated autoimmune hypoparathyroidism as the cause, as they could not detect significant titers of CaSR antibodies [3, 4]. With several authors demonstrating various pathophysiologies and the rarity of the incidence reported in our study, hypocalcemia should be thoroughly investigated before attributing it to ICPI-mediated parathyroid endocrinopathy.

Despite a good sample size with various cancers in our cohort, our study had several limitations, such as uneven distribution of ICPI drug type, retrospective nature of the study, and the high incidence of cases lost to follow-up. To reduce the bias from these limitations, we aim to follow up with a prospective study with an even distribution of ICPI drug type among the study population.

## 5. Conclusions

Our data suggest that the true hypocalcemia incidence after using albumin-corrected calcium values is very low in patients receiving ICPI, even in the presence of calcium altering factors. The percentage of patients with hypocalcemia is much higher and similar to the KEYNOTE-189 and CHECKMATE-067 trials when serum calcium values without albumin correction are used. Thus, the higher reported incidence of hypocalcemia in these trials is likely due to the reporting of serum calcium without albumin correction.

## Figures and Tables

**Table 1:** Baseline characteristics of the study population.

	Pembrolizumab (*n*)	Nivolumab (*n*)	Ipilimumab (*n*)	Nivolumab-ipilimumab (*n*)
Gender				
Male	49	99	3	6
Female	30	46	1	0

Cancer type				
Lung	85	85		
GI	6	9		
Melanoma	4	25	4	5
Neuroendocrine	1	4		
RCC	2	15		1
Bladder	5	2		
Head and neck	8			
Laryngeal	2			
Prostate	1			
Hodgkin lymphoma	1			
HCC		1		
Mesothelioma		2		

Factors affecting calcium levels				
Metastasis to bone	11	26		
Calcium supplements	6	10		1
Vitamin D	8	36	3	3
Bisphosphonates	9	9	1	
History of neck radiation	6			
History of parathyroidectomy				
>stage IIIB CKD		2		
History of hypo/hyperparathyroidism				

Lost to follow-up				
Deceased	15	25		
Disease progression	21	69		
Treatment completed	4	10	1	
Treatment changed	1			
Hospice	5	9		
Changed MD	1	2		
No-show	1			
Drug intolerance	3	4		
Ongoing	28	10		
Insurance		2		
Lost to follow-up		4		
Change in comorbidities		2		

## Data Availability

The data used to support the findings of this study are available from the corresponding author upon request.

## Abbreviations

ICPI: Immune checkpoint inhibitors
CKD: Chronic kidney disease
PD-1: Programmed cell death protein 1
PDL-1: Programmed cell death ligand 1
CTLA-4: Cytotoxic T-lymphocyte-associated protein 4
IRAE: Immune-related adverse event
CaSR: Calcium sensing receptor
CTCAE v 5.0: Common terminology criteria for adverse events version 5.0
PTHrP: Parathyroid hormone-related peptide
ECF: Extracellular fluid.

## References

[1] Buchbinder E. I., Desai A. (2016). CTLA-4 and PD-1 pathways. *American Journal of Clinical Oncology*.

[2] Stucci S., Palmirotta R., Passarelli A. (2017). Immune-related adverse events during anticancer immunotherapy: pathogenesis and management. *Oncology Letters*.

[3] Piranavan P., Li Y., Brown E., Kemp E. H., Trivedi N. (2019). Immune checkpoint inhibitor-induced hypoparathyroidism associated with calcium-sensing receptor-activating autoantibodies. *The Journal of Clinical Endocrinology & Metabolism*.

[4] Trinh B., Sanchez G. O., Herzig P., Läubli H. (2019). Inflammation-induced hypoparathyroidism triggered by combination immune checkpoint blockade for melanoma. *Journal for ImmunoTherapy of Cancer*.

[5] Win M. A., Thein K. Z., Qdaisat A., Yeung S.-C. J. (2017). Acute symptomatic hypocalcemia from immune checkpoint therapy-induced hypoparathyroidism. *The American Journal of Emergency Medicine*.

[6] Manohar S., Kompotiatis P., Thongprayoon C., Cheungpasitporn W., Herrmann J., Herrmann S. M. (2019). Programmed cell death protein 1 inhibitor treatment is associated with acute kidney injury and hypocalcemia: meta-analysis. *Nephrology Dialysis Transplantation*.

[7] Parent X., Spielmann C., Hanser A.-M. (2009). Calcémie corrigée: sous-estimation du statut calcique des patients sans hypoalbuminémie et des patients hypercalcémiques. *Annales de biologie clinique*.

[8] Rosen C. J., Compston J. E., Lian J. B. (2009). *Primer on the Metabolic Bone Diseases and Disorders of Mineral Metabolism*.

[9] Bushinsky D. A., Monk R. D. (1998). Calcium. *The Lancet*.

[10] Moore E. W. (1970). Ionized calcium in normal serum, ultrafiltrates, and whole blood determined by ion-exchange electrodes. *Journal of Clinical Investigation*.

[11] McLean F. C., Hastings A. B. (1934). A biological method for the estimation of calcium ion concentration. *Journal of Biological Chemistry*.

[12] McLean F. C., Hastings A. B. (1935). The state of calcium in the fluids of the body. I. The conditions affecting the ionization of calcium. *Journal of Biological Chemistry*.

[13] Pfitzenmeyer P., Martin I., d’Athis P. (2007). A new formula for correction of total calcium level into ionized serum calcium values in very elderly hospitalized patients. *Archives of Gerontology and Geriatrics*.

[14] Clarke B. L., Brown E. M., Collins M. T. (2016). Epidemiology and diagnosis of hypoparathyroidism. *The Journal of Clinical Endocrinology & Metabolism*.

